# Coping and positive mental health in Canada among youth and adults: findings from a population-based nationally representative survey

**DOI:** 10.24095/hpcdp.45.5.02

**Published:** 2025-05

**Authors:** Mihojana Jhumi, Laura L. Ooi, Karen C. Roberts, Melanie Varin

**Affiliations:** 1 Centre for Surveillance and Applied Research, Public Health Agency of Canada, Ottawa, Ontario, Canada; 2 Dalla Lana School of Public Health, University of Toronto, Toronto, Ontario, Canada

**Keywords:** self-rated mental health, happiness, life satisfaction

## Abstract

**Introduction:**

Coping is a protective factor for positive mental health (PMH) and an asset for population health. While there is evidence demonstrating a strong association between coping and PMH, less is known about how coping patterns differ across age groups. Given that age can impact coping ability, addressing this knowledge gap is warranted.

**Methods:**

We analyzed data from the 2019 Canadian Community Health Survey on the self-rated ability of adults and youth (N= 60 643; 12+ years) to cope with unexpected or difficult problems and day-to-day demands along with three PMH outcomes: self-rated mental health (SRMH), happiness and life satisfaction. All estimates were disaggregated by sociodemographic variables (sex, gender, household income quintile, immigration status, ethnocultural background, place of residence), stratified by five age groups, and age-specific regression analyses were conducted.

**Results:**

Prevalence of high coping varied by sex, gender, income, place of residence, immigration status and ethnocultural background. High coping was significantly associated with the three PMH outcomes across all age groups. Those with high coping were 4 to 6 times more likely to report high SRMH and high levels of happiness than those with lower coping. Individuals with high coping had a life satisfaction score between 0.84 and 1.32 units greater than individuals with lower coping.

**Conclusion:**

The consistent, positive relationship between high coping and PMH across all age groups provides valuable information for developing public health messaging and promotion efforts for adaptive coping to enhance population mental health.

**Highlights**

- In 2019, the prevalence of high coping was 69.6% to 86.3% across five age groups of youth and adults.
- The prevalence of high coping varied by sex, gender, income, immigration status, ethnocultural background and place (rural area or population centre) of residence.
- Of people with high coping, about three out of four reported high selfrated mental health and about four out of five reported high levels of happiness.
- The mean life satisfaction score was 8.3 (out of 10) for people with high coping.
- High coping increased the odds of high SRMH and happiness and was associated with higher mean life satisfaction for all groups.

## Introduction

The Public Health Agency of Canada (PHAC) defines positive mental health (PMH) as “the capacity of each and all of us to feel, think, act in ways that enhance our ability to enjoy life and deal with the challenges we face.”^1^ PMH has been recognized as contributing to Canada’s social and economic prosperity^2^ and alleviating risk factors for mental disorders; it can be associated with greater physical and mental health even in the presence of mental health problems.^3^,^4^ PMH is associated with reduced risk of mood and anxiety disorders,^5^ decreased symptom severity and better remission for patients with mental disorders^6^,^7^ and improved health and longevity in healthy populations.^8^

PHAC has been monitoring the PMH of adults (18+ years) and youth (12–17 years) in Canada through the Positive Mental Health Surveillance Indicator Framework (PMHSIF) since 2016.^9^,^10^ The PMHSIF provides routine estimates on a core set of PMH outcomes including self-rated mental health (SRMH), happiness, life satisfaction, psychological well-being and social well-being. The PMHSIF also provides information on risk and protective factors at the individual, family, community and societal levels.^9^,^10^

In order to identify populations and determinants that mental health promotion activities could target, PHAC also examines how patterns of PMH may vary across certain populations^3^,^11^-^13^ and explores relationships between PMH and different risk or protective factors.^14^-^16^

One individual-level protective factor is the ability to cope.^9^,^10^ Coping is defined as the cognitive and behavioural efforts to manage the internal and external demands of situations that are appraised as stressful.^17^,^18^ Coping can be active, where people try to modify the nature of stressful events through problem-solving, or it can be adaptive, where people regulate their emotional responses to these stressful events.^17^,^19^ According to the broaden-and-build theory,^20^ positive emotions can increase the capacity to engage in a variety of coping strategies in response to stress, which in turn build lasting social, physical and psychological resources that help to enhance well-being.^19^-^22^ Indeed, there is a considerable body of Canadian research that describes a significant association between coping and PMH outcomes, including psychological^23^,^24^ and emotional well-being.^3^,^25^

Studies identify age as a critical factor in the ability of individuals to cope with individual demands, pressures and challenges.^26^,^27^ Such coping requires adaptive skills that strengthen over time based on lived experience.^26^,^27^ In 2019, three out of four youth aged 12 to 17 years (75.5%) and four out of five adults (82.2%) in Canada reported having high levels of coping (or “high coping”).^10^ Further examination by age group reveals greater nuance. In 2019, the proportion of adults in Canada who reported high coping was highest among those aged 65 years and older (86.3%) and lowest among those aged 18 to 24 years (69.6%).^10^ Moreover, the extent to which adolescents and older adults use active coping to handle daily stressors has distinct implications for mental health outcomes.^28^ However, there is insufficient research examining coping patterns across different age groups and other pertinent sociodemographic variables in Canada.

For both youth and adults in Canada, coping varies by sociodemographic characteristics, including sex, income, place of residence and racialized population status.^10^ As such, monitoring PMH and coping across sociodemographic variables for each age group is crucial for understanding the health of the population and for identifying population groups that may benefit the most from public health interventions. In an effort to better describe and understand patterns of coping and associations with PMH, the objectives of this study were to:

- generate age group–specific national prevalence estimates of high coping among youth and adults, disaggregated by sociodemographic variables;
- provide prevalence estimates of three PMH outcomes, that is, high SRMH, high levels of happiness (or “high happiness”) and mean life satisfaction, among those with high coping; and
- examine age group–specific associations between coping and the three PMH outcomes.

## Methods

***Data and participants***

We analyzed data from the 2019 Canadian Community Health Survey (CCHS), which was designed to be a nationally representative, cross-sectional survey of people aged 12 or older across the 10 provinces in Canada.^29^ Excluded from this survey are individuals living on reserves or other Indigenous settlements, full-time members of the Canadian Armed Forces and individuals living in institutions, youth aged 12 to 17 years living in foster care and people living in the Rgion du Nunavik and Rgion des Terres-Cries-de-la-Baie-James Quebec health regions. The excluded populations represent less than 3% of the total population in Canada.

Respondents voluntarily completed the survey through computer-assisted personal interviews and computer-assisted telephone interviews.^29^ The response rate was 54.4%.^29^ Of the 65 970 survey respondents, 91.9% (n = 60 643) agreed to their data being shared with PHAC.^29^

***Measuring coping***

CCHS respondents were asked two questions:

(1) “In general, how would you rate your ability to handle unexpected and difficult problems, for example, a family or personal crisis?”

(2) “In general, how would you rate your ability to handle the day-to-day demands in your life, for example, handling work, family and volunteer responsibilities?”

There were four response options for each question: “excellent,” “good,” “fair” or “poor.” To align with the PMHSIF,^10^ participants were rated as having a high level of coping if they chose “excellent” for both questions, “excellent” for one question and “good” for the other, “good” for both questions or “excellent” for one and “fair” for the other. All other response options were classified as a lower level of coping. Statistics Canada has shown that reporting high levels of coping in response to these two questions is positively associated with other established, comprehensive measures of positive coping, such as the Ways of Coping scale,^30^ which was derived and modified from three coping scales.^31^

***Measuring PMH outcomes***

Three PMH outcomes (SRMH, happiness and life satisfaction) were included in this study and harmonized with the PMHSIF.^10^ To measure SRMH, CCHS respondents were asked if their mental health was “excellent,” “very good,” “good,” “fair” or “poor.” Those who chose “excellent” and “very good” were rated as having high SRMH.

To measure happiness, CCHS respondents were asked if they would usually describe themselves as “happy and interested in life,” “somewhat happy,” “somewhat unhappy,” “unhappy with little interest in life” or “so unhappy that life is not worthwhile.” Participants who chose “happy and interested in life” were categorized as having high happiness.

To measure life satisfaction, CCHS respondents were asked how they felt about their life “as a whole right now” on a scale of 0 (meaning “very dissatisfied”) to 10 (meaning “very satisfied”). Life satisfaction was treated as a numerical variable.

***Sociodemographic variables***

The data were stratified according to the following five age groups, in years: 12 to 17, 18 to 24, 25 to 44, 45 to 64 and 65 and older.^10^ We chose to use the following sociodemographic variables because they have been previously associated with coping or with PMH and could be potential confounders: sex, gender, household income distribution, immigration status, ethnocultural background and place of residence.^3^,^23^,^26^

**Sex and gender**

CCHS respondents were asked to report their sex at birth and their current gender using the response options “male” or “female” (or “please specify”) for both questions. We report on gender using the CCHS response options to maintain statistical rigour and for ethical purposes, despite these options being inconsistent with commonly accepted gender categories.^32^

**Household income**

Household income distribution was calculated using the adjusted ratio of total household income to low-income cut-off corresponding to the household and community size and categorized into quintiles.^29^

**Immigration status**

Immigration status was assessed using the derived immigrant flag variable that indicated whether a respondent was an immigrant or not. The immigrant category includes landed immigrants and non-permanent residents. Those who declared being born in Canada are considered non-immigrants.^29^

**Ethnocultural background**

We modified the “visible minority” variable developed by Statistics Canada to report on ethnocultural background. The variable used by Statistics Canada is based on the *Employment Equity Act*, which defines visible minorities as “persons, other than Aboriginal, who are non-Caucasian in race or non-white in colour.”^33^ The ethnocultural background categories used are “Arab/West Asian,” “Black,” “East/Southeast Asian,” “Indigenous,” “Latin American,” “South Asian” and “White.” Detailed information is available elsewhere.^29^,^33^

***Place of residence***

Respondents’ places of residence were derived from their postal code. Individuals living in continuously built-up areas with populations of at least 1000 and population densities of at least 400 per km^2^ were classified as living in population centres.^29^ The remaining respondents were classified as living in rural areas.

***Analysis***

We conducted descriptive and inferential analyses using statistical package SAS EG version 7.1 (SAS Institute Inc., Cary, NC, US). We estimated prevalence of high coping disaggregated across sociodemographic characteristics. We also estimated the prevalence of high SRMH and high happiness and the mean life satisfaction score among those with high coping. 

All analyses were stratified by the five age groups. We estimated coefficients of variation and 95% confidence intervals (CIs) using bootstrap weights (1000 replicates) provided by Statistics Canada. Age group–specific differences in sociodemographic breakdowns were established using unadjusted logistic regression (*p* < 0.05). To examine age group–specific associations between coping and PMH outcomes, we conducted regression analyses (unadjusted and adjusted for sociodemographic variables) for each age group. As the gender and sex variables were highly correlated with each other (i.e. multicollinearity), only sex was adjusted for in the regression models.

Logistic regression was conducted for high SRMH and high happiness, while linear regression was used for life satisfaction. For the regression analyses only, we used listwise deletion to address missing data. We chose this commonly used approach because the frequency of missing data was low and the sample sizes were large. For the logistic regression, odds ratios with 95% CIs that did not include 1.00 were considered statistically significant. For the linear regression, beta coefficients with 95% CIs that did not include 0 were considered statistically significant.

## Results

Most of the individuals in each age group lived in a population centre, were Canadian born and identified as White. The proportion of males and females was equally distributed across the five age groups ( Table 1).

**Table 1:** Sociodemographic characteristics of the overall population and stratified by age group, Canada (excluding territories), 2019

Sociodemographic characteristic	Proportion, %^ a^					
	Overall (N = 60 643)	12–17 years (n = 3609)	18–24 years (n = 2999)	25–44 years (n = 13 572)	45–64 years (n = 15 549)	65+ years (n = 24 914)
Sex						
Male	49.4	51.2	53.3	49.6	49.4	46.5
Female	50.6	48.8	46.7	50.4	50.6	53.5
Gender						
Male	49.4	51.3	53.0	49.6	49.4	46.4
Female	50.6	48.7	47.0	50.4	50.6	53.6
Household income adequacy quintile						
Q1 (lowest)	20.2	20.0	28.5	19.5	15.2	25.2
Q2	19.9	21.9	19.4	20.1	16.2	24.8
Q3	20.8	22.0	19.7	22.5	19.6	19.9
Q4	19.1	21.0	17.1	19.3	21.8	15.1
Q5 (highest)	20.0	15.1	15.3	18.5	27.3	15.1
Place of residence						
Population centre	82.8	81.4	86.7	86.3	80.8	78.9
Rural area	17.2	18.6	13.3	13.7	19.2	21.1
Immigration status						
Yes	27.8	15.4	23.6	31.7	27.4	28.6
No	72.2	84.6	76.4	68.3	72.6	71.4
Ethnocultural background						
White	72.3	62.4	58.0	65.9	77.4	85.1
South Asian	5.6	7.1	8.1	7.8	4.0	2.6
East/Southeast Asian	8.5	11.7	13.9	9.8	7.0	5.0
Black	3.5	5.8	5.7	4.4	2.9	1.4
Arab/West Asian	2.3	3.0^ E^	3.3^ E^	3.1	2.0	1.0^ E^
Latin American	1.6	1.5^ E^	2.9^ E^	2.6	1.0	0.5^ E^
Indigenous	3.6	6.0	4.7	3.9	3.5	1.7

Source: Canadian Community Health Survey – Annual Component.^29^

**Abbreviation**: Q, quintile. 

^a^ All estimates are weighted. 

^E^ Estimate should be interpreted with caution due to high sampling variability (coefficient of variation between 15.1% and 35%). 

Over four out of five (81.4%) respondents reported having high coping (Table 2). The 18- to 44-year cohorts had significantly lower prevalence of high coping than the 45- to 64-year and older cohorts, from 69.6% for the 18- to 24-year cohort to 86.3% for the 65 years and older cohort. A similar pattern was seen across PMH outcomes, where we saw lower PMH among the younger cohorts than the older ones. Most of the respondents also reported high SRMH (from 55.6% to 72.4%) and high happiness (from 66.7% to 77.8%). Mean life satisfaction scores ranged from 8.0 to 8.7, with similar scores across all the adult age groups and the highest score in the 12- to 17-year cohort.

**Table 2:** Coping and PMH outcomesa in the overall population and stratified by age group, Canada (excluding territories), 2019

Coping and PMH outcomes	Overall (N = 60 643)	12–17 years (n = 3609)	18–24 years (n = 2999)	25–44 years (n = 13 572)	45–64 years (n = 15 549)	65+ years (n = 24 914)
Coping, % (95% CI)						
High	81.4 (80.9–82.0)	75.5 (73.4–77.5)	69.6 (66.9–72.3)	79.5 (78.5–80.6)	85.5 (84.6–86.4)	86.3 (85.5–87.1)
Low	18.6 (18.0–19.1)	24.5 (22.5–26.6)	30.4 (27.7–33.1)	20.5 (19.4–21.5)	14.5 (13.6–15.4)	13.7 (12.9–14.5)
Self-rated mental health, % (95% CI)						
High	67.1 (66.4–67.8)	72.4 (70.1–74.6)	55.6 (52.7–58.5)	64.4 (63.2–65.7)	69.8 (68.6–71.0)	71.2 (70.2–72.2)
Low	32.9 (32.2–33.6)	27.6 (25.4–29.9)	44.4 (41.5–47.3)	35.6 (34.3–36.8)	30.2 (29.0–31.4)	28.8 (27.8–29.8)
Happiness, % (95% CI)						
High	75.5 (74.9–76.0)	75.6 (73.6–77.6)	66.7 (64.0–69.3)	74.7 (73.5–75.8)	77.5 (76.5–78.6)	77.8 (76.9–78.8)
Low	24.5 (24.0–25.1)	24.4 (22.4–26.4)	33.3 (30.7–36.0)	25.3 (24.2–26.5)	22.5 (21.4–23.5)	22.2 (21.2–23.1)
**Mean life satisfaction score^ b^**	8.1 (8.1–8.1)	8.7 (8.6–8.8)	8.0 (7.9–8.0)	8.1 (8.1–8.2)	8.0 (8.0–8.1)	8.2 (8.1–8.2)

Source: Canadian Community Health Survey – Annual Component.^29^

**Abbreviations**: CI, confidence interval; PMH, positive mental health. 

**Note**: Some percentages may not sum to the exact total due to rounding. 

^a^ All estimates are weighted. 

^b^ Life satisfaction was rated on a scale from 0 (very dissatisfied) to 10 (very satisfied). 

Across all age groups except the 45- to 64-year age group, there were significant sex and gender differences in the prevalence of high coping (Table 3). Compared with females, males reported a significantly greater prevalence of high coping. Adults aged 25 years and older in the highest household income adequacy quintiles had significantly greater prevalence of high coping than those in the lowest income group. There were no significant differences in coping associated with income among youth (12–17 years) and young adults (18–24 years).

**Table 3:** Overall and age group–specific prevalence estimates of high coping disaggregated by sociodemographic variables, Canada (excluding territories), 2019

Sociodemographic characteristic	Proportion of respondents with high levels of coping, % (95% CI)^ a^					
	Overall (N = 60 643)	12–17 years (n = 3609)	18–24 years (n = 2999)	25–44 years (n = 13 572)	45–64 years (n = 15 549)	65+ years (n = 24 914)
Sex						
Male (reference)	83.2 (82.4–84.0)	78.8 (75.9–81.8)	73.8 (70.0–77.6)	81.5 (80.0–83.1)	86.3 (85.1–87.5)	88.1 (87.0–89.2)
Female	79.7^ *^ (78.9–80.6)	72.0^ *^ (68.9–75.0)	64.9^ *^ (61.1–68.6)	77.6^ *^ (76.1–79.2)	84.7 (83.4–86.0)	84.8^ *^ (83.7–85.9)
Gender						
Male (reference)	83.2^ *^ (82.4–84.0)	78.7 (75.8–81.7)	74.0 (70.3–77.8)	81.5 (80.0–83.1)	86.3 (85.1–87.6)	88.1 (87.0–89.2)
Female	79.8^ *^ (79.0–80.6)	72.2^ *^ (69.2–75.2)	65.0^ *^ (61.2–68.8)	77.6^ *^ (76.1–79.2)	84.7 (83.4–86.0)	84.8^ *^ (83.7–85.9)
Household income quintile adequacy						
Q1 (lowest) (reference)	76.6^ *^ (75.3–78.0)	74.8 (69.6–80.1)	68.3 (63.0–73.6)	75.0 (72.5–77.6)	78.3 (75.9–80.8)	82.5 (80.8–84.2)
Q2	80.5 (79.2–81.9)	74.9 (69.8–80.0)	67.9 (61.3–74.5)	80.7^ *^ (78.5–82.9)	83.0^ *^ (80.6–85.5)	84.6 (82.8–86.3)
Q3	82.4 (81.1–83.6)	75.3 (70.9–79.8)	68.3 (62.0–74.7)	80.7^ *^ (78.3–83.1)	86.9^ *^ (85.0–88.8)	88.2^ *^ (86.6–89.9)
Q4	83.2 (82.0–84.4)	76.6 (72.3–80.9)	69.9 (63.6–76.2)	80.9^ *^ (78.5–83.3)	87.4^ *^ (85.5–89.2)	89.4^ *^ (87.6–91.1)
Q5 (highest)	84.4^ *^ (83.2–85.5)	73.8 (68.4–79.3)	75.3 (69.9–80.6)	80.3^ *^ (77.8–82.7)	88.4^ *^ (86.8–89.9)	89.6^ *^ (87.7–91.4)
Place of residence						
Population centre	81.0^ *^ (80.4–81.7)	75.2 (72.7–77.6)	68.8^ *^ (65.8–71.7)	79.2^ *^ (78.0–80.3)	85.5 (84.5–86.6)	86.1 (85.2–87.1)
Rural area (reference)	83.3 (82.4–84.3)	76.9 (73.3–80.5)	74.9 (70.1–79.7)	81.9 (79.9–83.9)	85.3 (83.8–86.8)	86.8 (85.5–88.1)
Immigration status						
Yes (reference)	82.0 (80.7–83.3)	75.8 (69.1–82.4)	68.6 (62.6–74.6)	82.0 (80.0–84.1)	85.3 (83.3–87.4)	84.0 (82.0–86.1)
No	81.2 (80.6–81.9)	75.7 (73.4–77.9)	70.0 (67.0–73.1)	78.4^ *^ (77.2–79.7)	85.5 (84.5–86.4)	87.2^ *^ (86.4–87.9)
Ethnocultural background						
White (reference)	82.4 (81.8–83.0)	74.8 (72.3–77.3)	71.4 (68.3–74.5)	79.6 (78.4–80.7)	85.8 (84.8–86.7)	87.0 (86.2–87.7)
South Asian	81.1 (78.4–83.8)	84.5^ *^ (77.0–92.0)	69.1 (59.2–79.0)	81.4 (77.0–85.7)	87.1 (81.9–92.3)	80.6 (71.9–89.4)
East/Southeast Asian	76.1^ *^ (73.3–78.9)	76.9 (70.2–83.6)	59.8^ *^ (50.5–69.1)	76.4 (71.6–81.1)	83.9 (79.7–88.1)	81.2^ * ^ (75.1–87.2)
Black	81.8 (78.2–85.4)	80.2 (70.5–90.0)	72.2 (60.5–83.9)	84.0 (78.8–89.1)	83.3 (75.4–91.1)	87.7 (79.1–96.4)
Arab/West Asian	80.0 (75.3–84.6)	68.6 (53.5–83.6)	70.8 (56.5–85.2)	83.0 (77.1–89.0)	87.2 (77.7–96.7)	68.6^ E^ (47.3–89.8)
Latin American	81.5 (75.7–87.4)	86.4^ *^ (75.3–97.5)	60.9 (40.2–81.5)^ E^	87.8 (81.5–94.2)	81.6 (70.1–93.1)	81.1 (60.2–100.0)
Indigenous	74.6^ *^ (71.8–77.3)	60.4^ *^ (52.0–68.9)	74.5 (66.2–82.9)	70.8^ *^ (65.8–75.8)	80.9^ *^ (76.8–85.0)	86.4 (82.4–90.3)

**Source**: Canadian Community Health Survey – Annual Component.^29^

**Abbreviations**: CI, confidence interval; Q, quintile.

^a^ All estimates are weighted. 

^E^ Interpret with caution due to high sampling variability (coefficient of variation between 15.1% and 35%). 

* Significantly different from the reference group, at *p* < 0.05. 

Adults aged 18 to 44 years (but none of the other age groups) living in rural areas had a significantly greater prevalence of high coping than those living in population centres. Immigrants aged 25 to 44 years reported a significantly greater prevalence of high coping than non-immigrants in the same age group (82.0% vs. 78.4%). The reverse occurred among adults aged 65 years and older, with non-immigrants reporting a significantly greater prevalence of high coping than immigrants (87.2% vs. 84.0%). Indigenous youth (12–17 years) and adults (25–64 years) reported a significantly lower prevalence of high coping than non-Indigenous people in the same age groups. Young adults (18–24 years) and older adults (65+ years) who identified as White (71.4% and 87.0%, respectively) had a significantly greater prevalence of high coping than those who identified as East or Southeast Asian (59.8% and 81.2%, respectively). Youth (12–17 years) who identified as Latin American (86.4%) or South Asian (84.5%) had a significantly greater prevalence of high coping than those who identified as White. There were no other significant differences in the prevalence of high coping in racialized populations within the age groups.

Approximately three out of four individuals with high coping reported having high SRMH and about four out of five reported having high levels of happiness (Figures 1A and 1B). Mean life satisfaction score among those with high coping varied between 8.2 (for adults aged 45–64 years) and 8.9 (for youth aged 12–17 years). In age groups with high coping, youth (12–17 years) had the highest prevalence of high SRMH (80.3%) and adults aged 45 to 64 years had the highest prevalence of high happiness (83.1%). Young adults aged 18 to 24 years had the lowest prevalence of high happiness (77.4%) and high SRMH (68.2%).

**Figure 1A:**
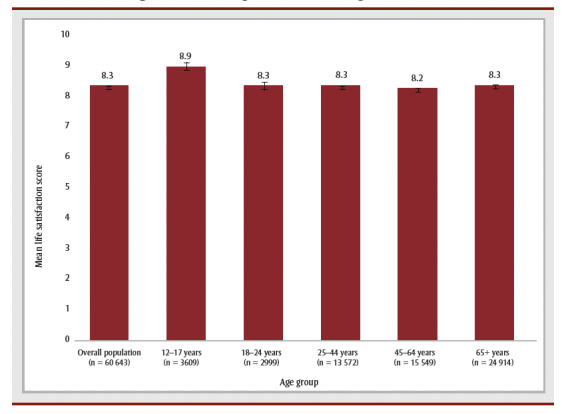
Overall and age-specific estimates of mean life satisfactiona among those with high levels of coping, Canada (excluding territories), 2019

**Figure 1B:**
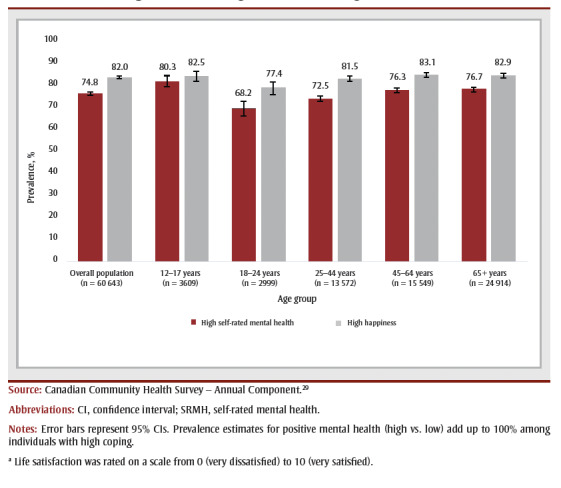
Overall and age-specific estimates of high SRMH and happiness among those with high levels of coping, Canada (excluding territories), 2019

Coping was robustly associated with all three PMH outcomes in both the unadjusted and adjusted analyses across all age groups (see Table 4). Individuals who reported having high coping were between four and six times more likely to report high SRMH (adjusted odds ratio [aOR] between 4.2 and 6.5) and high happiness (aOR between 3.8 to 5.3) than those with lower coping. Similarly, individuals with high coping had a mean life satisfaction score between 0.8 and 1.32 units greater than individuals with lower coping. There were minimal differences in the PMH effect estimates across the age groups. The percentage change between the unadjusted and adjusted analyses was less than 10% for all three PMH outcomes.

**Table 4:** Age group–specific logistic and linear regression models of the association between high levels of coping and PMH outcomes, Canada (excluding territories), 2019

Age group, years	High SRMH		High happiness		Life satisfaction score	
	OR (95% CI)	aOR (95% CI)	OR (95% CI)	aOR (95% CI)	β (95% CI)	aβ (95% CI)
Overall	5.71 (5.28–6.18)	5.54 (5.11–6.01)	4.72 (4.36–5.11)	4.57 (4.20–4.96)	1.16 (1.10–1.22)	1.16 (1.10–1.22)
12–17	4.43 (3.42–5.73)	4.23 (3.20–5.57)	3.79 (2.91–4.93)	3.80 (2.87–5.04)	0.87 (0.72–1.02)	0.85 (0.71–0.98)
18–24	5.82 (4.50–7.54)	5.99 (4.55–7.90)	4.27 (3.26–5.60)	4.60 (3.44–6.16)	1.07 (0.90–1.25)	1.06 (0.89–1.23)
25–44	5.22 (4.55–5.99)	5.09 (4.42–5.85)	4.67 (4.08–5.35)	4.61 (4.00–5.31)	1.10 (1.00–1.19)	1.06 (0.97–1.15)
45–64	6.58 (5.64–7.67)	6.54 (5.56–7.68)	5.46 (4.68–6.37)	5.29 (4.49–6.23)	1.36 (1.22–1.50)	1.28 (1.14–1.41)
65+	5.58 (4.85–6.43)	5.39 (4.68–6.21)	3.93 (3.43–4.51)	3.93 (3.41–4.53)	1.37 (1.24–1.49)	1.32 (1.19–1.45)

**Source**: Canadian Community Health Survey – Annual Component.^29^

**Abbreviations**: aOR, adjusted odds ratio; aβ, adjusted beta coefficient; β, beta coefficient; CI, confidence interval; OR, odds ratio; PMH, positive mental health; SRMH, self-rated mental health. 

**Notes**: The reference group were individuals who reported having a low level of coping. All associations were statistically significant at *p* < 0.05. Covariates adjusted for included sex, age group (only for the overall population), household income adequacy quintile, place of residence, immigrant status and ethnocultural background. 

## Discussion

Coping is a critical determinant of PMH. It influences people’s emotional responses and ability to manage stress in challenging situations. Although there is evidence of a relationship between coping and PMH,^23^,^24^ no studies have investigated this association across different age and sociodemographic groups in Canada.

***Overall and age group–specific prevalence of high coping and by sociodemographic variables***

Older adults (65+ years) had the highest prevalence of coping while youth (12–17 years) and young adults (18–24 years) had the lowest prevalence. A possible explanation is that coping skills take time to develop. Transitioning into adulthood is accompanied by major uncertainty and life-changing decisions involving education, career development, family formation, relocation and more.^34^ These novel obstacles help develop the enhanced coping skills seen in older adults.

Across all age groups, except the 45- to 64-year cohort, more males than females reported high coping, with the difference widest among young adults aged 18 to 24 years. The literature also reports that, compared with males, females tend to experience higher rates of chronic stress and daily stressors that can negatively impact their sense of control and adaptive coping capacity.^35^ However, caution is warranted before drawing firm conclusions as these results could potentially be explained by sex differences in stress appraisal. Indeed, findings from a meta-analysis suggest that women tend to appraise stress more severely than men.^36^ As the coping measure used in this study focussed on perceived ability to handle stress, the observed differences could be due to how males and females appraised the question, rather than true differences in coping. Exploring this further would be worthwhile.

Among adults aged 25 years and over, the prevalence of high coping was significantly greater for those in the highest household income adequacy quintile. The ability to adopt effective and healthy coping strategies is associated with socioeconomic status, which determines the availability of resources and expectations of control a person has when navigating daily stressors.^37^ Of note, our findings indicate no significant differences in the prevalence of high coping between those in the highest household income quintile and those in the lowest quintiles in the youngest cohorts (aged 12–17 and 18–24 years). This suggests that income is not a major influence on coping for younger populations as much as it is for older age groups. One reason that could be that the stressors associated with income and associated expectations of control may be felt differently by adolescents and young adults.^38^

Adults aged 18 to 44 years and living in rural areas reported a significantly greater prevalence of high coping than those living in population centres. This may be indicative of differences in sense of community belonging, which has a significant relationship with mental health.^39^ For example, in Ontario, rural residents reported a stronger sense of community belonging than did residents of population centres; sense of community belonging is associated with better coping and resiliency when faced with unfavourable circumstances.^40^ This finding is also consistent with national estimates examining inequalities in high community belonging.^10^

Compared with non-immigrants, immigrants aged between 25 and 44 years reported higher levels of coping, whereas immigrants aged 65 years and older reported lower levels of coping. A potential explanation for these mixed results is suggested by the decline in the healthy immigrant effect and in immigrants’ mental health with more time spent in the destination country.^41^ The greater prevalence of high coping among older non-immigrants compared to immigrants could also be attributed to migration-related stressors such as social isolation, lack of English or French language proficiency and limited access to culturally sensitive health care.^42^,^43^ These, in turn, can lead to older adult immigrants experiencing greater difficulties coping with familial, societal and personal changes from pre-migration contexts.^44^

Our study found that youth aged 12 to 17years and adults aged 25 to 44 years who identify as Indigenous had a significantly lower prevalence of high coping than those who did not identify as Indigenous. These findings are supported by other evidence of discrepancies in PMH outcomes.^45^ These discrepancies may be due to the disproportionately high number of barriers Indigenous people encounter in accessing mental health care services,^46^ which make it difficult to acquire the tools and support individuals may need to cope. These systemic barriers are rooted in cultural discontinuity, discrimination and the intergenerational cycle of trauma perpetuated by colonization that continues to affect the Indigenous peoples’ healing and coping.^47^,^48^ What was unexpected was that this discrepancy did not occur among young adults aged 18 to 24 years or adults aged 45 years and older. Further investigation of age-associated stressors is needed to fully capture the complexity of coping among Indigenous youth and adults.

Of note, the only difference in prevalence of high coping in racialized populations was among those aged 25 to 44 years. This finding could be due to the heterogeneity of the sample and differences in how individuals with diverse ethnocultural backgrounds conceptualize coping and daily stressors.^49^,^50^

***Prevalence of PMH indicators among those with high coping***

Overall, those who reported high levels of coping had a substantially greater prevalence of high PMH. The prevalence of high SRMH and mean life satisfaction was highest among youth aged 12 to 17 years, while the prevalence of high happiness was highest among adults 45 years and older. Young adults (18–24 years) had the lowest prevalence of high SRMH and high happiness.

These findings are not surprising and conform with those shown in the PMHSIF.^10^ The age group–related variations in PMH may, in part, be due to differences in developmental stage coping mechanisms when solving everyday problems.^51^ For instance, a measurement burst study of participants aged 20 to 79 years found that older adults were more likely than younger ones to report particular events as less unpleasant or severe.^28^ Our findings supports the socioemotional selectivity theory, which asserts that older adults are more motivated to engage in positive, emotionally meaningful experiences and implement proactive coping to minimize exposure to adverse stressors.^28^,^52^

It is important to take into account the unique challenges associated with transitioning from adolescence to adulthood that requires distinct coping strategies and resources. The current study results could help support the promotion of targeted public health strategies, such as public health messaging and development of stress-management resources in various settings (e.g. educational institutions, workplaces, households, etc.) to foster positive coping across the developmental stages.^53^

***Age group–specific associations between coping and three PMH measures***

Our findings show a strong relationship between coping and the three PMH outcomes across the five age groups. Our results provide support for the broaden-and-build theory^20^ and are consistent with previous cross-sectional^3^ and longitudinal^24^ Canadian research that demonstrated a prominent association between coping and PMH. As decreases in population-level PMH were documented during the COVID-19 pandemic,^54^,^55^ it will be important to continue monitoring coping to assess if it continues to be impacted.

***Strengths and limitations***

To our knowledge, this is the first study to report age group–specific prevalence estimates of coping across different sociodemographic variables and to examine age group–specific associations between coping and PMH. This type of stratification enabled us to detect similarities and differences between subgroups that would not have been captured by analyzing the overall sample. Because the survey data were collected over 12 months, seasonality is not a concern in terms of effects on participant responses.

Coping is a complex construct that encompasses a variety of behaviours and strategies (e.g. active coping, disengagement, restraint coping, emotion-focussed coping and others).^56^ The coping measure used in this study was high level and could not capture those nuances. To better identify the distinctions between the different coping behaviours and strategies, future studies could replicate this analysis using more detailed coping scales (such as the Ways of Coping scale).^30^,^31^

Due to the cross-sectional nature of data collection, causality and temporality between any of the variables cannot be established. The response rate to the 2019 CCHS was only 54.4%, which increases the likelihood of sampling bias. It is possible that certain populations (e.g. those experiencing mental ill-health) were less likely to opt in. In addition, it is likely that the sociodemographic characteristics of individuals who did not opt into the survey differ from those who did, resulting in non-response bias. While we tried to address this through survey weighting, it is an important limitation to note. Evidence from the National Household Survey suggests that there is a higher risk of non-response bias for immigrants, Indigenous people, some racialized groups and educational status (non-response bias indicators from −3.4 to +7.3 for these groups).^57^ While we were unable to find non-response bias indicator estimates for the CCHS, we expect similar estimates given the analogous methodology.

There could also be methodological differences in how different age groups interpret and respond to the coping and PMH measures. As we did not conduct measurement invariance analyses across age groups, caution is warranted when interpreting our findings. It should also be noted that the study sample and results are not inclusive of individuals living in the territories or on First Nations reserves and other Indigenous communities. Moreover, the survey was available to respondents who speak English or French, which limits the representation of some populations. Lastly, all data in the analysis were self-reported and are subject to social desirability bias.

## Conclusion

The current study reveals that, depending on the age group, the prevalence of high coping varies by sex, gender, income, place of residence, immigration status and ethnocultural background. Moreover, high coping substantially increased the likelihood of reporting high SRMH, happiness and life satisfaction for every age group. These findings address a gap in the current public health surveillance evidence by providing empirical support linking an individual-level determinant (coping) and PMH, offering insights for policy makers promoting the mental well-being of people in Canada. As our study did not examine indicators of psychological and social well-being, further exploration of the relationship between coping and different PMH outcomes among children, youth and adults is encouraged.

## Acknowledgements

The authors would like to thank Natalie Gabora (Public Health Agency of Canada) for reviewing the manuscript. We would also like to thank all Canadian Community Health Survey (CCHS) participants.

## Funding

None.

## Conflicts of interest

The authors have no conflicts of interest to disclose.

## Authors’ contributions and statement

MJ: Formal analysis, methodology, project administration, visualization, writing – original draft, writing – review and editing.

LLO: Conceptualization, methodology, writing – review and editing. 

KCR: Writing – review and editing.

MV: Conceptualization, formal analysis, methodology, validation, project administration, writing – review and editing.

The content and views expressed in this article are those of the authors and do not necessarily reflect those of the Government of Canada.
